# Implementation of lung ultrasonography by general practitioners for lower respiratory tract infections: a feasibility study

**DOI:** 10.1080/02813432.2024.2343678

**Published:** 2024-05-20

**Authors:** Félix Amiot, Thomas Delomas, François-Xavier Laborne, Thomas Ecolivet, Richard Macrez, Axel Benhamed

**Affiliations:** a Emergency Department-SAMU50, Centre Hospitalier Mémorial Saint-Lô, Saint-Lô, France; b SAMU91, Centre Hospitalier Sud-Francilien, Corbeil-Essonnes, France; c Primary Care Centre, Troarn, France; d Normandie University, UNICAEN, INSERM, U1237, PhIND "Physiopathology and Imaging of Neurological Disorders," Institut Blood and Brain @ Caen-Normandie, Caen, France; e Department of Emergency Medicine, Caen University Hospital, Caen, France; f Emergency Department-SAMU69, Centre Hospitalier Universitaire Edouard-Herriot, Hospices Civils de Lyon, Lyon, France

**Keywords:** General practice, primary care, family medicine, point-of-care ultrasound, lung ultrasonography

## Abstract

**Objective:**

To evaluate the feasibility of lung ultrasonography (LUS) performed by novice users’ general practitioners (GPs) in diagnosing lower respiratory tract infections (LRTIs) in primary health care settings.

**Design:**

A prospective interventional multicenter study (December 2019–March 2020).

**Settings and subjects:**

Patients aged >3 months, suspected of having LRTI consulting in three different general practices (GPs) (rural, semirural and urban) in France.

**Main outcome measures:**

Feasibility of LUS by GPs was assessed by (1) the proportion of patients where LUS was not performed, (2) technical breakdowns, (3) interpretability of images by GPs, (4) examination duration and (5) patient perception and acceptability.

**Results:**

A total of 151 patients were recruited, and GPs performed LUS for 111 (73.5%) patients (LUS group). In 99.1% (*n* = 110) of cases, GPs indicated that they were able to interpret images. The median [IQR] exam duration was 4 [3–5] minutes. LRTI was diagnosed in 70.3% and 60% of patients in the LUS and no-LUS groups, respectively (*p* = .43). After LUS, GPs changed their diagnosis from ‘other’ to ‘LRTI’ in six cases (+5.4%, *p* < .001), prescribed antibiotics for five patients (+4.5%, *p* = .164) and complementary chest imaging for 10 patients (+9%, *p* < .001). Patient stress was reported in 1.8% of cases, 81.7% of patients declared that they better understood the diagnosis, and 82% of patients thought that the GP diagnosis was more reliable after LUS.

**Conclusions:**

LUS by GPs using handheld devices is a feasible diagnostic tool in primary health care for LRTI symptoms, demonstrating both effectiveness and positive patient reception.

**Trial Registration Number:**

Clinicaltrial.gov: NCT04602234, 20/10/2020.

## Introduction

Lower respiratory tract infections (LRTIs) are a frequent reason for consultations in general practice (GP) [1]. Imaging is a component of the diagnostic strategy, and it has been shown that chest computed tomography (CT) is more accurate than conventional chest radiography in diagnosing LRTI [2–4]. However, healthcare costs, radiation exposure and the availability of this exam prevent it from being part of the daily routine and diagnosis strategy for every patient with suspected LRTI [5,6]. Conversely, lung ultrasonography (LUS) is a noninvasive, safe and inexpensive imaging exam whose implementation is increasing within primary care settings, notably due to its miniaturization [7–9]. In addition, point-of-care ultrasound (POCUS) has a short learning curve, including LUS [10–12]. It has been described as the ‘stethoscope of the future’ [13] and could play a role in primary care alongside traditional clinical examination and symptom appraisal [14]. It is highly accurate in detecting pneumothorax, pleural effusion, pulmonary contusions and pneumonia [15–19]. In addition, LUS has been shown to improve the accuracy of LRTI diagnosis in emergency departments, where its use has become standard practice [20,21]. LUS has also been assessed for clinically suspected community-acquired pneumonia. It holds the potential to be implemented into the routine toolkit of general practitioners (GPs) and this integration could lead to a decrease in the prescription of chest imaging and referrals to secondary care [22–24]. A number of primary care physicians consider implementing POCUS in their practices as an add-on to the traditional clinical assessment of patients [10]. However, feasibility and impact of LUS in such a setting have been insufficiently explored [22]. There is specifically a lack of data regarding patient experience and acceptability [25], and these points should be addressed before widespread implementation of POCUS in primary care settings. The objective of the present study was therefore to assess the feasibility and the impact of using LUS in primary care for patients presenting with clinically suspected LRTI as well as evaluate patients’ perception and acceptability.

## Materials and methods

### Study design and setting

The study was designed as a prospective, interventional, multicenter feasibility study in France. It was conducted in office-based GPs. Patients were recruited from 1 December 2019 to 31 March 2020 by 15 different GPs during their respective working hours (Appendix 1).

### GPs recruitment and training

The recruitment strategy for GPs was developed to maximize the extrapolation of the findings across a spectrum of environments, by deliberately extending invitations to practices located in urban, semi-urban and rural locales. This was achieved by proactively engaging with GPs from various backgrounds, without limiting participation to those affiliated with academic institutions. Special attention was given to GPs who had previously shown an interest in research. GPs were not familiar with POCUS in their daily practice, and none had received ultrasound training in the past. Prior to recruitment of patients, a 3-h transthoracic ultrasonography (US) training course was delivered by an expert sonographer and his/her team with direct teaching. The training course started with a theoretical session dedicated to device use and rationale for image acquisition, pathological findings, and common LUS patterns and their interpretation. GPs were taught about the sonographic features of infection, effusion, pneumothorax and interstitial alveolar syndrome. They were also trained to distinguish pneumonia from bronchitis US imaging patterns. Another session consisted of observing scans, scanning under supervision, and training in reporting. The training program for this study did not include any pre-existing e-learning materials or prior instruction.

In the absence of a dedicated lung ultrasound protocol explicitly validated for the primary care setting [23], the methodology for image acquisition was predicated upon a standardized eight-point examination. This approach adheres to the international evidence-based recommendations for point-of-care lung ultrasound [16]. GPs were systematically instructed in the application of the BLUE protocol – a rigorously validated framework for lung ultrasound [26].

Trainees all used the same U-Lite™ handheld ultrasound device (Sonoscanner, Inc., Paris, France) during the training period and the study. Image acquisition was performed with a low-frequency 2–5 MHz convex probe.

### Participants

All patients aged >3 months presenting to their GP with symptoms of an acute LRTI were included. There were no exclusion criteria. Suspicion of LRTI was based on GPs judgment and symptoms compatible with LRTI included: cough, shortness of breath, chest pain or discomfort, wheezing, fever and/or sputum production.

### Outcome measures

The primary outcome measure was the feasibility of LUS performed by GPs in patients presenting with symptoms of acute LRTI (pneumonia or bronchitis). This composite outcome was evaluated according to the following criteria: (1) the proportion of patients where LUS was not performed; (2) the number of technical breakdowns; (3) the proportion of images that could be interpreted by GPs; (4) timing; (5) patient perception as well as acceptability.

Secondary outcomes included the clinical influence on patient management (antibiotics and imaging test prescription, hospital referral after LUS), and hospitalizations within seven days of follow-up.

### Data collection

GPs were first requested to complete a standardized case report form (CRF) for collecting data pertaining to patient demographics (age, sex), main reason for consultation (symptoms), clinical findings, initial medical diagnosis, prescriptions (antibiotics, imaging) and discharge status (ambulatory or hospital referral). Subsequent to the initial consultation, GPs were free to perform or not perform a standardized eight-point LUS. If they did so, they were asked to describe ultrasound images and report the timing involved in performing the exam. They were also required to disclose any modifications to their initial diagnosis and patient management (therapeutic, additional imaging and discharge status). Therapeutic changes following LUS that could degrade initial patient care (e.g. cancel a biological or imaging test prescribed before the LUS was performed) were not allowed.

Patients undergoing LUS had a post consultation follow-up by telephone at day 7. A physician (FA) collected the following information: symptoms evolution (improvement, persistence or worsening). If applicable, imaging results (chest CT scan or radiograph) and the hospital discharge summary were also compiled in the CRF by GPs who routinely received these documents. This approach enabled the identification of any discrepancies between the conclusions drawn from the additional medical records and those reached during the initial GP consultation. In a separate questionnaire, information regarding patient perceptions and experience relative to LUS was collected, and the following questions were asked: ‘Was LUS uncomfortable or painful?’ ‘Was LUS stressful?’ ‘Did LUS help to better understand the diagnosis?’ ‘Do you feel more confident with your GP diagnosis thanks to LUS?’ ‘Do you think that integrating LUS into the daily GP routine could be of help?’

### Statistical analysis

Baseline characteristics were described by median and interquartile range [IQR] for continuous variables and by frequency and percentage for categorical variables. Proportions (%) were calculated among those with data. Comparison of categorical variables was performed by a McNemar test or an exact Fisher test, and the Wilcoxon rank test was used for continuous variables. All tests were conducted bilaterally with a first alpha species risk of 5%. Statistical analyses were performed by using R^®^ software (version 3.5.0) (R Foundation for Statistical Computing, Vienna, Austria).

### Ethics approval and consent to participate

This study was approved by a French Ethics Committee (Commission de Protection des personnes, CPP Sud Méditerranée 16/12/2019) and was retrospectively registered at Clinicaltrial.gov (NCT04602234, 10/20/2020). In accordance with French law, written informed consent was obtained from all included patients. GPs provided written consent to participate in the study. Data were anonymized, and the investigators were blinded to the identity of the participating patients as well of GPs. The study was conducted in accordance with the ethical principles for medical research described in the Declaration of Helsinki.

## Results

### Feasibility and patient characteristics

**Figure 1.**
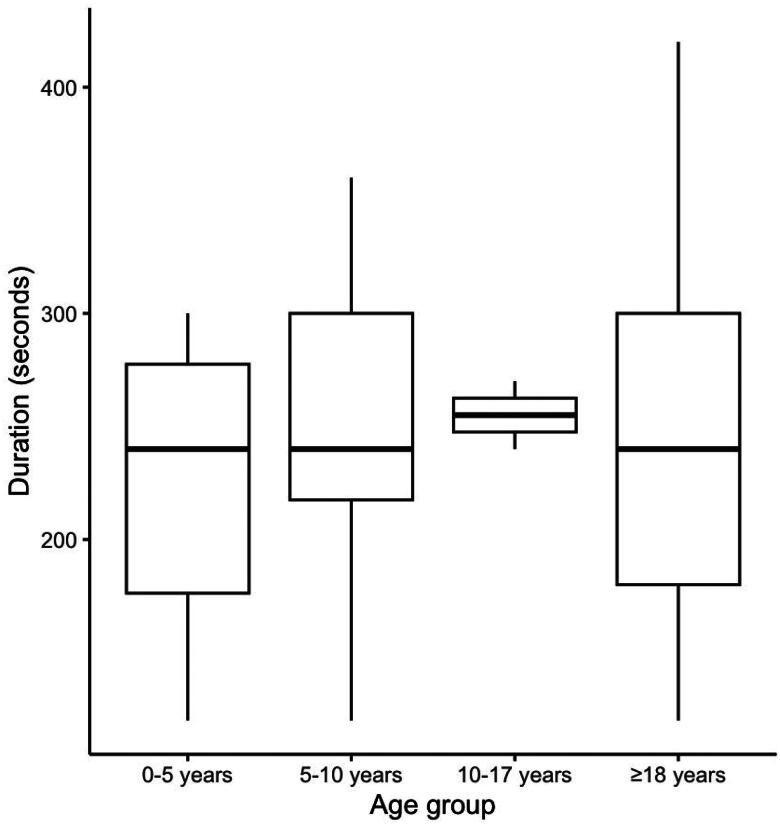
Distribution of LUS duration (in seconds) across age groups.

A total of 151 patients were recruited. Among them, GPs performed LUS for 111 (73.5%) patients (LUS group). No patient refused to participate to the study. In 99.1% (*n* = 110) of cases, GPs indicated that they were able to interpret LUS images. One GP failed to interpret because of technical issues. The median [IQR] exam duration was 240 [180–300] seconds, and there was no clinically significant difference across age groups (Figure 1). In the LUS group, GPs observed the following pathological ultrasonographic features: unilateral B-lines (*n* = 19), consolidation (*n* = 10), bilateral B-lines (*n* = 5) and pleural effusion (*n* = 4). Compared to patients who did not receive LUS, those who did were younger (59.1 [29.4–73.7] years vs. 70.6 [42.9–88] years, *p* < .001) and more frequently male (61.3% vs. 42.5%, *p* = .044). In the LUS group, age ranges from 2 to 106 years old. The main reason for consultation (cough or dyspnea) was not different between the two groups (*p* = .068). A total of 70.3% and 60% of patients were found to have an LRTI in the LUS group and no-LUS groups, respectively (*p* = .43). Although the difference was not statistically significant, there was a clinically significant higher proportion of patients who received an antibiotic in the LUS group (55% vs. 37.5%, *p* = .07). Conversely, a greater proportion of patients who had a LUS were prescribed a chest radiograph (24.3% vs. 5%, *p* < .001; Table 1).

**Table 1.** Patient characteristics and initial management.

	Total population, *n* = 151	LUS group, *n* = 111 (73.5)	No-LUS group, *n* = 40 (36.5)	*p* Value
Age, median [IQR], years	61.9 [35.4–75.5]	59.1 [29.4–73.7]	70.6 [42.9–88]	<.001
Sex, male	85 (56.3)	68 (61.3)	17 (42.5)	.044
Main reason for consultation				.068
Cough	108 (71.5)	84 (75.7)	24 (60)	
Dyspnea	43 (28.5)	27 (24.3)	16 (40)	
GPs geographical area of practice				<.001
Rural	38 (25.2)	15 (13.6)	23 (57.5)	
Semirural	43 (28.5)	35 (31.5)	8 (20)	
Urban	70 (46.4)	61 (54.9)	9 (22.5)	
Initial diagnosis				.43
LRTI	102 (67.5)	78 (70.3)	24 (60)	
Other diagnosis^a^	49 (32.5)	33 (29.7)	14 (40)	
Antibiotic prescription	76 (50.3)	61 (55)	15 (37.5)	.07
Imaging test prescription	31 (20.5)			<.001
Chest radiograph	29 (19.2)	27 (24.3)	2 (5)	
Chest CT-Scan	2 (1.3)	2 (1.8)	0 (0)	
Hospital referral	2 (1.3)	1 (0.9)	1 (2.5)	.46

CT: computed tomography; GP: general practitioner; IQR: interquartile range; LRTI: lower respiratory tract infection; LUS: lung ultrasound.

^a^ Other diagnoses included nose-throat-ear infection and acute heart failure.

### Impact of LUS on patient management

Table 2 depicts the impact of LUS on patient management. After the exam, GPs changed their diagnosis from ‘other’ to ‘LRTI’ in six cases (+5.4%, *p* < .001; four were initially diagnosed with upper respiratory tract infection while two with unspecified chest pain), prescribed antibiotics for five more patients (+4.5%, *p* = .164), and complementary chest imaging for 10 patients (+9%, *p* < .001). There was no significant difference in baseline patient characteristics among patients whose initial diagnosis or management was refined after LUS (age: 59.9 [34.8–74.1] vs. 57.6 [22.8–69.6] years, *p* = .24; male sex: 75% vs. 64.5%, *p* = .83 in the groups without and with modification). Similarly, there was no significant difference in the exam time (4 [3–5] vs. 4.5 [4–5] minutes, *p* = .35) or in the initial diagnosis (LRTI: 76.3% vs. 74.2%, *p* = .81).

**Table 2.** Impact of LUS on patient management.

	Before LUS	After LUS	Delta Δ (%)	*p* Value
LRTI diagnosis	78 (70.3)	84 (75.7)	+6 (5.4)	<.001
Antibiotic prescription	61 (55)	66 (59.5)	+5 (4.5)	.164
Imaging test prescription	29 (26.1)	39 (35.1)	+10 (9)	<.001
Hospital referral	1 (0.9)	1 (0.9)	0 (0)	1

LRTI: lower respiratory tract infection; LUS: lung ultrasound.

### Patient experience

A total of 109 (98.2%) patients in the LUS group responded to the patient experience survey. Of them, 89.9% (*n* = 98) had never experienced LUS in primary care before, none reported any discomfort or pain during the exam, 1.8% (*n* = 2) felt extra stress, 81.7% (*n* = 89) declared that they had a better understanding of the diagnosis after LUS, 82.6% (*n* = 90) thought that the GP diagnosis was more reliable and 95.4% (*n* = 104) believed that the LUS device could be a useful tool for GP practice (Table 3).

**Table 3.** Patient experience survey responses.

	Yes	No	Don’t know
Did you have any previous experience of POCUS in primary care?	10 (9.2)	98 (89.9)	1 (0.9)
Did you feel any discomfort or pain during the exam?	0 (0)	109 (100)	0 (0)
Was the exam an extra source of stress for you?	2 (1.8)	106 (97.2)	1 (0.9)
Did you better understand the diagnosis thanks to the LUS?	89 (81.7)	8 (7.3)	12 (11)
Did you think that the diagnosis was more accurate thanks to the LUS?	91 (83.4)	5 (4.6)	13 (11.9)
Do you believe that LUS could be a beneficial tool for your GP?	104 (95.4)	2 (1.8)	3 (2.8)

LUS: lung ultrasound; POCUS: point-of-care ultrasound.

### Patient follow-up

A total of 81.1% (*n* = 90) of patients could be followed up and contacted at day 7. Among them, 90% (*n* = 80) reported improvement, 6.7% (*n* = 6) reported persistence and 3.3% (*n* = 3) reported worsening of their symptoms. Among those who had an imaging test prescription, 51.6% (*n* = 16) underwent the exam, the final diagnosis was in accordance with the initial GP diagnosis for all of them. For patients who were hospitalized within the first seven days following their consultation with a GP (*n* = 5), the final diagnoses noted in the hospital discharge summaries were consistent with the initial diagnoses made by the GPs for all these cases.

## Discussion

### Summary

In this multicenter trial, we found that LUS can be easily implemented in primary care after a short training session. Participant GPs recruited three patients out of four meeting inclusion criteria and declared that they were able to interpret LUS imaging in almost all cases. In addition, exams could be completed in less than 5 min in most cases. We also found that patient experience and acceptability were both excellent.

### Comparison with existing literature

In France, the mean consultation length is 16 min in line with most developed countries, with a maximum of 21 min in the U.S.A. and 21.5 min in Sweden [27]. Therefore, LUS may be easily integrated into GP practice based on the present study, which showed that in 75% of cases, the time needed to complete the exam was 5 min or less with no significant differences across age groups. This aligns with the findings of Aakjær Andersen et al. where the median time taken to perform LUS was 5 min [24]. Another interesting point is that we found no significant difference in patient age between those included in the LUS group and those who did not undergo LUS. This suggests that LUS could be implemented for both pediatric and older adult populations in primary care settings routine. Claes et al. demonstrated that LUS is a fast and feasible technique to detect lung consolidation in children suspected of pneumonia. With its high negative predictive value, the authors concluded that it could substitute for radiographs in excluding lung consolidation, thus reducing radiation exposure, which is a major concern, especially in this vulnerable population [28]. However, given the number of participating GPs and the expected seasonal surge in LRTI during winter, we anticipated enrolling a larger number of patients. We therefore cannot exclude the possibility that GPs underreported the number of eligible patients, particularly among the youngest children and babies. Herein, the youngest child included was 2 years old and only 6% of patients (*n* = 9) were under the age of 5. Various factors may have contributed to the lower inclusion rates observed in our study including academic activities and other professional duties beyond their primary practice. Additionally, some GPs focused part of their practice on specialized areas like sports medicine or gynecology, leading to a patient demographic that may not fully match our study’s inclusion criteria. In terms of sex, we noted that a smaller proportion of females were included in the LUS group. This may be attributable to anticipated difficulty in acquiring images due to anatomical issues (women breast). Potential challenges to perform LUS in children and female patients warrant further investigations. The findings reported by Andersen et al. based on an online survey highlighted other avenues to explore, including potential barriers to the implementation of POCUS, such as the lack of remuneration and high workload [29].

Although very few patients had prior experience with POCUS, a large majority of them declared that LUS contributed to a better understanding of their disease and increased GP diagnosis reliability. Having patients become directly involved in their own healthcare by visualizing LUS images probably contributed to these findings. This is of great interest for primary care since it could increase medication compliance as previously suggested by Kerse et al. who showed through a survey with telephone follow-up that physician–patient concordance and patient trust were significantly related to compliance [30]. In addition, no patient had discomfort or pain, and only two individuals experienced additional stress. In line with the present findings, Danish authors also reported a very high level of acceptability by patients. More specifically, a majority (82%) of patients felt POCUS gave them a better understanding of their health problem, made them feel more secure (86%) and increased their trust in the physician’s assessment (65%). In this study, POCUS use also improved the level of service (95%) and the quality of care (94%) in GP according to surveyed patients [25].

Regarding the influence of LUS on diagnosis and patient care, some modifications were observed in less than 10% of cases, which is a smaller proportion than reported in a previous prospective observational study by Andersen et al. However, authors explored the impact of POCUS in the setting of GP, focusing on GPs who have used POCUS for a minimum of six months. Their findings highlighted a significant impact of LUS use, with adjustments observed in diagnosis, management strategies or treatment plans in 59%, 52% and 34% of the cases, respectively [24]. These findings suggest that LUS may be of help in patient management. However, authors did not explore if those changes improved patient care, or if it caused harm in terms of false positive findings, misdiagnosis, overdetection and potential, subsequent overtreatment which warrant for future research to address this issue.

### Strengths and limitations

A major strength of the present study relies on the characteristics of the participating GPs, offering substantial potential for the generalizability of our findings. Notably, all GPs were native users of POCUS. The gender distribution among the GPs was balanced, with an equal number of male and female participants. Furthermore, the GPs represented a wide array of practice settings, spanning from rural to urban settings. This point is particularly pertinent, as the use of LUS for diagnosing LRTI could be beneficial in low-resource settings, such as rural communities where access to imaging services can be limited [31]. Although there were disparities in the number of patients included according to the geographical area of the GPs’ practices, our results suggest the feasibility of implementing LUS in rural and semi-rural settings. Nonetheless, future research should specifically target these environments to delve deeper into the preliminary findings presented herein.

Some limitations must be acknowledged. First, the interpretation of images was not overseen by an expert; therefore, errors may have occurred. Furthermore, recent findings have indicated that the 8-zone protocol may not provide as accurate results in children as an extended scan protocol does [31]. Nevertheless, we mainly aimed to evaluate LUS feasibility and not GP performance. Second, this was not a randomized trial, and GPs were not asked to provide information pertaining to the reason for not performing LUS. This point will need further investigation to better understand GP needs. Third, given that GPs were not allowed to degrade their initial management, we were unable to fully evaluate the effects of implementing LUS, but a reduction in the use of irradiating exams and inappropriate antibiotic prescriptions may be expected. Fourth, we provided GPs with handheld devices that are known to be affordable, but whether GPs would be willing to use standard US devices in their practice is questionable. Fourth, a possible patient underreporting cannot be excluded as well as the existence of a selection bias, particularly notable in the pediatric population.

### Implications for research and/or practice

Although further studies are warranted to evaluate GP LUS learning curves and its impact on the reduction of inappropriate medications, the present study’s findings, and the emergence of a new generation of clinicians who have become familiar with POCUS may enhance the widespread adoption of LUS in primary care in the future.

## Conclusions

This study showed that LUS performed by GPs with handheld devices can be easily integrated into the management of ambulatory patients presenting with LRTI symptoms. Positive patient experience and high acceptability warrant the success of LUS implementation in primary care settings. However, there is a need for further investigation into potential barriers, including increased workload, remuneration issues, the cost of equipment, and requirements for continuing education. Additionally, future research should evaluate the overall impact of POCUS on patient management within primary care environments.

## Appendix 1. GP investigator characteristics

**Table:**

	*n* = 15
Age, median [IQR], years	42 [35.5–50]
Sex, male	8 (53.3)
Experience, years	9 [5.5–19]
0–10	4 (26.7)
10–15	1 (6.7)
15–20	2 (13.3)
>20	8 (53.3)
GPs geographical area of practice	
Rural	4 (26.7)
Semirural	4 (26.7)
Urban	7 (46.7)
Previous experience of POCUS	0 (0)

GP: general practitioner; POCUS: point-of-care ultrasound.
